# Examining Quality of Work Life in Atlantic Canadian Long-Term Care Homes: Protocol for a Cross-Sectional Survey Study

**DOI:** 10.2196/66338

**Published:** 2025-03-31

**Authors:** Janice M Keefe, Rose McCloskey, Marilyn J Hodgins, Caitlin McArthur, Adrian MacKenzie, Lori E Weeks, Carole A Estabrooks

**Affiliations:** 1 Department of Family Studies and Gerontology Mount Saint Vincent University Halifax, NS Canada; 2 Nursing and Health Sciences University of New Brunswick, Saint John Saint John, NB Canada; 3 Faculty of Nursing University of New Brunswick Fredericton, NB Canada; 4 Department of Physiotherapy Faculty of Health Dalhousie University Halifax, NS Canada; 5 Health Workforce Planning Nova Scotia Department of Health and Wellness Government of Nova Scotia Halifax, NS Canada; 6 Department of Community Health & Epidemiology Faculty of Health Dalhousie University Halifax, NS Canada; 7 School of Health Administration Faculty of Health Dalhousie University Halifax, NS Canada; 8 Faculty of Nursing University of Alberta Edmonton, AB Canada

**Keywords:** residential long-term care, care staff, Atlantic Canada, quality of work life, work environment, health and well-being

## Abstract

**Background:**

The Canadian long-term care (LTC) workforce cares for increasingly complex residents. With greater care needs come greater demands. Despite this, LTC staffing and resources are largely unchanged and underresearched over the last decade. The Atlantic provinces are home to the oldest population in Canada, indicating a high need for LTC. The health and well-being of the LTC workforce are critical components of care quality, yet only in Western Canada are such data routinely and systematically collected. Translating Research in Elder Care is a 2-decade research program studying the LTC work environment and has found strong links between the working conditions of LTC staff and resident outcomes. We draw upon their success to generate the evidence needed to understand, support, and manage the LTC workforce in Canada’s four Atlantic provinces.

**Objective:**

This study aims (1) to assess the quality of work life among staff in LTC homes in Atlantic Canada; (2) to examine the effects of the work environment on the quality of work life; and (3) to build capacity for research in the LTC sector in Atlantic Canada among knowledge users, researchers, and trainees. The objective of this paper is to describe the approach needed to examine the quality of work life and health of care staff in LTC homes.

**Methods:**

Stratified random sampling will be used to recruit homes in Atlantic Canada. The sampling frame was designed to recruit 25% of the LTC homes in each of the 4 provinces with proportional representation by size; ownership model; and, if applicable, region or language. Key outcome variables include measures of mental health and well-being, quality of work life, intention to leave, workplace context, and missed or rushed care. Primary data will be obtained through structured interviews with care aides and web-based surveys from registered nurses, licensed practical nurses, managers, and allied health providers. Eligible participants were from an LTC home with at least 25 residents, 90% of whom were aged 65 years or older, and had worked in the home for at least 3 months. Multivariate analyses include regression analysis for explaining predictors of quality of work-life outcomes and multilevel modeling for more complex relationships of staff outcomes by provinces and LTC home characteristics.

**Results:**

Data collection and cleaning are complete as of October 2024 (N=2305). Care aides (n=1338), nurses (n=724), allied health providers (n=154), and managers (n=89) from 53 homes make up the sample. Data analysis is ongoing. Initially, individual reports will present descriptive data for each participating LTC home. Concurrent analysis is planned for publication in peer-reviewed journals.

**Conclusions:**

This peer-reviewed research protocol lays the foundation for a comprehensive analysis of the effects of the work environment on the quality of work life of LTC staff in Atlantic Canada.

**International Registered Report Identifier (IRRID):**

DERR1-10.2196/66338

## Introduction

### Background

This protocol is for a collaborative investigation that aims to address profound deficiencies in data to understand, support, and manage the long-term care (LTC) workforce in Canada’s 4 Atlantic provinces. In Canada, LTC homes are funded by provincial governments and provide a range of health and personal care services to predominantly older adults who require 24-hour nursing, personal, and support care [1]. Residents of LTC have higher levels of acuity and have more complex care needs than in the past decade as a result of increased life expectancy and delayed entry due to an increased emphasis on home care [2]. Approximately 81% of residents in Canadian LTC homes live with mild to severe cognitive impairment, 27.1% have seven or more chronic conditions, and 51.5% have extensive limitations in activities of daily living [3]. As the care needs of residents become greater, so do the demands placed on the LTC workforce. Despite this, LTC staffing and resources have remained largely unchanged and underresearched over the last decade [4,5].

The LTC workforce cares for residents with complex needs, but their work environments are strained by heavy workloads, inadequate resources, and critical staffing shortages [4,5]. The health and well-being of the LTC workforce are critical components of care quality [5], and yet only in the Western Canadian provinces of British Columbia, Alberta, and Manitoba are such data routinely and systematically collected [6]. In Atlantic Canada, no data are available on workforce characteristics or the work experiences of LTC care providers (ie, indicators of health, well-being, or burnout). LTC in Canada is provincially regulated, meaning policies, regulations, and practices differ among provinces; fundamental differences in how LTC is organized between Western provinces and Atlantic Canada influence how staff work and care for residents, including staffing models and ratios, and education and training requirements of staff. While many other Canadian provinces have continuously relied on internationally trained care workers to supplement the domestic workforce, this is a relatively new phenomenon in the Atlantic Canadian provinces of Nova Scotia, Prince Edward Island, Newfoundland and Labrador, and New Brunswick. Chamberlain et al [7] reported in 2018 that only 35% of care aides working in Alberta LTC homes spoke English as a first language in comparison to 95% of LTC care staff in a Nova Scotian 2021 pilot study [8]. Additionally, according to the 2021 census, between 96% to 99% of the population in Nova Scotia, Prince Edward Island, Newfoundland and Labrador spoke English as a first language, and as a bilingual province, nearly 70% of the New Brunswick population spoke English as a first language while the remaining 30% spoke French [9]. While recent efforts from governments in Atlantic Canada to bring internationally educated workers into LTC have been largely deemed successful from a recruitment perspective [10-12], little is known about how the changing composition of the workforce impacts the LTC environment. More specifically, it is unknown if, and to what degree, domestic and international staff feel supported as they adjust to a more diverse workplace. Further, the combined population of all 4 Atlantic provinces is fewer than 2.5 million [13] and far fewer staff are trained internationally. These differences limit the ability to generalize workforce data from the Western provinces to Atlantic Canada.

Given the relationship between work and care conditions [14,15], data on the LTC workforce are important as they allow policy makers to identify and address workforce issues that compromise resident care and the health and well-being of care staff. Understanding the work environment will assist in future workforce planning and the strategies designed to improve work conditions and ultimately care conditions.

### Translating Research in Elder Care

Translating Research in Elder Care (TREC) is a program of research spearheaded by Carole Estabrooks and a group of researchers from Alberta Canada who have been studying the LTC work environment in the Western Canadian provinces for nearly 2 decades [6]. TREC has analyzed the impact of the LTC work environment on staff quality of work life, mental health, and well-being outcomes in 7 waves of data collection [5,7,16-18]. These data enable in-depth longitudinal analysis that demonstrates the impacts of policy changes, interventions, and events such as COVID-19 [19-21]. The TREC research program also links the staff survey data within a clinical microsystem to resident outcomes using data from the Resident Assessment Instrument for LTC [22]. Evidence of strong links between the working conditions of LTC staff and resident outcomes is reported. For example, significant relationships were found between the use of best practices and care staff’s social capital and organizational slack (time and staffing) [23]. In examining the demographic profile of the LTC workforce, staff who provide the majority of direct resident care had little formalized training, were racially diverse, and had high rates of English as a second language [7]. More recently, TREC has designed and delivered interventions to enhance the work environment in LTC, such as Safer Care for Older Persons in (residential) Environments [21] and Improving Nursing Home care through Feedback on Performance [24], and a coherent breathing intervention to decrease stress, insomnia, and anxiety [25].

The TREC measurement system, including the care aide structured interview and the regulated staff web-based survey, were consistent with the instruments used in this study in Atlantic Canada. The minor exceptions were because of localized staff job titles and translation of survey instruments to enable staff in designated French LTC homes in New Brunswick to participate in their French language. The goal of our team is to build upon the work of TREC and to use their comprehensive data collection tools to build an Atlantic Research Collaboration (ARC) and to generate the evidence needed to understand, support, and manage the LTC workforce in Canada’s 4 Atlantic provinces. Ultimately, we will identify work environment areas that are amendable to intervention so that targeted interventions can be designed and delivered at the regional, provincial, institutional, and unit levels to enhance the quality of the LTC work environment.

### Study Purpose and Objectives

This ARC on LTC (ARC LTC) involved researchers and collaborators form 4 provinces to describe and examine the relationship between the health and quality of work life of LTC staff and their work environment (organizational context). The research aims are as follows.

- To assess the quality of work life among staff in LTC homes in Nova Scotia, Prince Edward Island, Newfoundland and Labrador, and New Brunswick.
- To examine the effects of the work environment on quality of work life.
- To build capacity for research in the LTC sector in Atlantic Canada among knowledge users, early career researchers, and trainees.

## Methods

### Study Design

This is a cross-sectional, multilevel survey study with an integrated knowledge translation approach with knowledge users (ie, representatives from LTC homes, health authorities, provincial ministries, and key sector organizations) embedded throughout the project. We use the Checklist for Reporting Results of Internet E-Surveys (CHERRIES) reporting guidelines for web-based or internet surveys as a framework for our approach [26]. A detailed description using the CHERRIES checklist is present in Multimedia Appendix 1. Our methods are consistent with the established recruitment and data collection protocols used by TREC [23,27] and will enable comparison of data not only across the Atlantic Canadian provinces but also with the Western Canadian provinces.

### Study Context

The LTC sectors in Atlantic Canada are diverse with variations in names assigned to LTC homes, regulatory oversight, ownership models, and titles given to unregulated care staff (Table 1). The study’s design is sensitive to these differences with some modifications made to the sampling frame to reflect the uniqueness of individual provinces. In this protocol, we use the terms LTC home and care aide to describe the facility where care staff work or residents live, and the unregulated staff, respectively.

**Table 1:** Characteristics of homes by province in 2023.

Characteristics	Nova Scotia	New Brunswick	Newfoundland and Labrador	Prince Edward Island	
Name of LTC^a^ homes	Nursing home	Nursing home	LTC homes	Manors or nursing homes	
Government responsible	Department of Seniors and LTC	Department of Social Development	Department of Health and Community Services	Department of Health	
Legislative framework	Homes for Special Care Act	Nursing Home Act	Health and Community Care Services Act	Community Care Facilities and Nursing Homes Act	
Ownership model	Public, private for-profit, and not-for-profit	Private corporations run by boards of directors	Public	Public, private for-profit, and not-for-profit	
Title for unregulated care staff or care Aides	Continuing care assistant	Resident aide	Personal care attendant	Resident care worker	
Regulated nursing staff	Registered nurses and licensed practical nurses	Registered nurses and licensed practical nurses	Registered nurses and licensed practical nurses		Registered nurses and licensed practical nurses
Allied health providers presence of these providers varies by province and LTC home	Recreation therapist, aide social worker, dietitian physiotherapist, aide occupational therapist, and aide	Recreation therapist, aide social worker, dietitian physiotherapist, aide occupational therapist, and aide	Recreation therapist, aide social worker, dietitian physiotherapist, aide occupational therapist, and aide		Recreation therapist, aide social worker, dietitian physiotherapist, aide occupational therapist, and aide

^a^LTC: long-term care.

### Theoretical Framing

The critical socioecological framework [28] is used to guide this research. This framework depicts how behaviors are influenced by characteristics of, and interactions between, the individual and immediate social network (microsystem), local environment or community (mesosystem), and the larger system (macrosystem). The ecological framework supports the bidirectionality of this influence (eg, the work environment may affect individual staff feelings of well-being and vice versa). The framework highlights the complexity of staff’s experiences and agency within the LTC environment and their ability to engage in change to enhance resident care. It acknowledges how the work environment is shaped and buffered by factors in the micro, macro, and meso levels. Embedding our research in the critical socioecological framework will enable us to examine the inter-relationships among individual experiences, the work environment, and the larger provincial and regional LTC policy context.

We will examine LTC at the macro, meso, and micro levels (see Multimedia Appendix 2 [29-39] for how the survey content is organized at the meso and micro levels). At the macro level, all 4 Atlantic provinces provide public funding to LTC. Although regulations, guidelines, and staffing ratios differ by province, individual homes vary in how they operationalize them internally. At the meso level, institutional practices, policies, and work culture, along with heavy workloads associated with complex LTC residents have a major impact on staff. About 80% of resident care in Canada is provided by unregulated care aides who have little, if any, formal education [7]. Despite their pivotal role in LTC, care aides may not receive the recognition they deserve, nor do they always feel like valued members of care teams [23]. Contextual factors in work environments such as leadership, culture, and social capital, influence individual staff at the micro level and account for greater job satisfaction, empowerment, and autonomy [40,41]. The critical socioecological framework can facilitate our understanding of the multilayered influencers on LTC staff’s quality of work life, and ultimately, resident care and quality of life in LTC homes in Atlantic Canada.

The dynamic nature of the LTC environment including things such as changes in the complexity of resident care needs and the increasing number of staff who speak English as a second language, could prompt changes in workplace policies and culture. Changes in both policies and culture can impact how staff work at the micro level as well as how organizations operate at the meso level. The nature of these bidirectional relationships affects how staff work, how they experience their work, and their perceived ability to support the changes needed in the work environment.

### Sampling

#### Facility Sampling and Eligibility

A stratified random sampling technique will be used to recruit homes from each of the 4 provinces. The sampling frame was designed to recruit 25% of the LTC homes in each province (Table 2) with proportional representation by size (large: >120 beds; medium: 70-119 beds; and small: 25-69 beds); ownership model (eg, public, not-for-profit, and private for-profit); and, if applicable, region or language. These strata have been previously associated with residents’ quality of care [42,43]. Oversampling will be done in Newfoundland and Labrador and Prince Edward Island to ensure a minimum of 8 homes in these provinces and a sufficient sample to enable cross-tabulation. Because Prince Edward Island only has 1 health region, and New Brunswick is a bilingual province with designated English and French homes, the region strata will not be used in Prince Edward Island, and linguistic designation will be used in New Brunswick.

To be eligible to participate, homes need to provide 24-hour, on-site housing and health care services for older adults by professional (nursing) staff, be stand-alone facilities (ie, not attached to an acute care hospital), and have a minimum of 25 LTC beds with at least 90% of the resident population aged 65 years or older. Homes will be ineligible if they are hybrid care homes integrated with an acute care facility or hospital, or share central services (eg, human resources and laundry) with an acute care facility or hospital.

**Table 2:** Number of LTC^a^ homes included in the sample and strata by province (2023 estimates).

Characteristics	Nova Scotia	New Brunswick	Newfoundland and Labrador	Prince Edward Island	Total
Total number of LTC homes, n	92	73	43	19	227
Eligible homes, n (% of total)	81 (88%)	71 (97.3%)	27 (62.8%)	15 (78.9%)	194 (85.5%)
Sample (25% of eligible), n	20	18	8^b^	8^b^	54
Strata	Region ownership size	Language size	Region size	Ownership size	—^c^

^a^LTC: long-term care.

^b^Newfoundland and Labrador and Prince Edward Island were oversampled.

^c^Not applicable.

#### Staff Sample and Eligibility

Our sample will include senior administrators, managers, regulated nursing staff, unregulated care staff, and allied health providers (Table 3). All staff must have worked at the LTC home for 3 months or longer. In addition to the 3-month criteria, managers must work a minimum of 50% of the time at the LTC home, nurses must work a minimum of 6 shifts a month at the LTC home, care aides must be able to identify a unit they work on more than 50% of the time and work a minimum of 6 shifts a month on that unit, and allied health must provide at least one-third of their services that equals 6 days a month at the LTC home. Senior administrators who hold executive positions in LTC, such as chief executive and operation officers, will be required to provide the standardized facility (eg, physical design) and unit-level data (eg, staffing models).

**Table 3:** Estimated number of care staff in LTC^a^—both regulated and unregulated by province.

Characteristics	Nova Scotia	New Brunswick	Newfoundland and Labrador	Prince Edward Island	Total by staff group
All registered nurses or licensed practical nurses in LTC, n	1611	2625	1278	299	5813
Sample pool from LTC homes (25%), n	322	656	320	150^b^	1448
Desired sample (60% of eligible staff), n	193	394	192	90	869
All care aides, n	3866	4875	962	710	10,413
Sample pool from LTC homes (25%), n	773	1219	241	355^b^	2588
Desired sample (60% of eligible staff), n	464	731	144	213	1553
Desired care staff (registered nurses, licensed practical nurses, or care aides) sample total, n	—^c^	—	—	—	2422

^a^LTC: long-term care.

^b^Prince Edward Island was oversampled (50%).

^c^Not applicable.

#### Sampling Procedures

With the exception of Prince Edward Island, each province will have a team of staff responsible for recruitment and data collection. Due to the size and resources in Prince Edward Island, recruitment and data collection will be assigned to the Nova Scotia team. Each provincial research team will create a database of their LTC homes that includes information on each stratum, along with the name and contact information of facility administrators. The stratified proportional random sampling procedure will be performed centrally by a project manager who will then inform provincial teams of the randomly selected LTC homes to recruit. Homes identified in the randomization procedure will be emailed a letter of invitation from the province’s lead investigator and if necessary, a follow-up phone call from a member of the research team. Meetings via Microsoft Teams will be held with homes who express interest in the study. During these meetings, the nature and purpose of the study will be explained along with details of what participation entails. Homes that agree to enroll in the study will be asked to sign a facility agreement form that outlines the expectations of participation. If approval of an operator is required for facility participation the operator is asked to sign an operational approval form prior to the home signing a facility agreement.

Once a home agrees to participate, research staff will conduct orientation meetings (one in-person visit and MS Team or telephone meetings as needed) with administrators and site liaisons to (1) verify the eligible participant pool for completing staff surveys; (2) identify the number and types of care units, staff assignment, and shifts; and (3) discuss the logistics of data collection (eg, dates for data collection, promotion and recruitment strategies, interview flow and schedule, contact information for a site liaison).

### Measures

Data will be collected using TREC’s suite of survey instruments. TREC data collection tools have been administered in Western Canada at several points in time [5,7,18,23] and in Nova Scotia in 2021 with 10 LTC homes [8]. A key component of the surveys is the Alberta Context Tool (ACT), which was developed to measure 10 dimensions of the organization context within health care settings (eg, leadership, culture, feedback, staffing). The ACT contains slight variations for each category of staff and was developed and refined for use in LTC [44]. In addition to the ACT, other surveys will be used to collect data on staff demographics, their perceptions of physical and mental health, well-being, burnout, job satisfaction, as well as missed and rushed care. Permission to access and use these tools has been obtained. Minor revisions to the survey instruments have been made with permission to reflect Atlantic Canada–specific context (eg, provincial term used for care aides). Details of these tools, including their psychometric properties, are outlined in Multimedia Appendix 2 [29-39].

### Translation

Many of TREC's suite of tools are only available in English, which means they have to be translated to French to accommodate the New Brunswick French LTC homes. The quality of these translations is critical in maintaining the integrity of the data collected, and the comparability of data across provinces. The TREC research team has a preexisting rigorous translation protocol that was modified by Hoben et al [45] and is based on international best practices [46]. The first step in the process is to identify the proprietary rights of each survey and to establish which tools are available in French. Of the tools in the suite, 6 tools have existing French versions, and 2 of these cannot be altered. We will obtain permission to translate the remaining 6 tools from English to French (Multimedia Appendix 2).

Translation of these 6 tools and the full TREC survey will involve a rigorous systematic process of forward translation from English to French, back translation from French to English, and cognitive debriefing. Once the forward and back translation processes are completed, additional reviewers will examine the changes for accuracy and precision and, if necessary, make recommendations for the final translation.

We will recruit 4 LTC staff members to complete the translated surveys to verify the understanding, clarity, and appropriateness of the items. This step is for language, comprehension, and cultural issues only; these pilot data will not be included in the study’s sample.

Additional translations will also be required for other research materials, such as demographic questionnaires, consent forms, standardized instructions, and explanations of items within individual surveys.

### Data Collection Procedures

Based on TREC’s experience, data to determine care home units for the study and the number of eligible staff will be collected during the initial in-person meeting between a member of the research team and an LTC care home administrator. Data collection for staff will not begin until these data are obtained. Meetings will take approximately 1 hour and will include a tour of the LTC facility and will allow research staff to build a relationship with care home administration.

A staff member at each care home will be designated as the site liaison by the LTC administrator. The site liaison will work with research staff on promotion, participant recruitment, and scheduling of data collection. An individualized data collection schedule will be cocreated for each home that allows for maximum access to eligible staff and minimum disruptions to the home. Data collection will take place during days, evenings, nights, and weekends. Data collection will be scheduled over a 1-week period, but, if necessary, more time will be provided.

Informed consent will be obtained from all staff prior to data collection. Staff survey data will be collected through a self-administered web-based survey or during a structured interview using Microsoft Teams. Regulated staff will be provided with a link to the closed web-based survey and instructed to complete it voluntarily and independently on the web using the Nooro research platform, on which the survey was thoroughly tested by project staff prior to data collection. Codes given to staff can be used to leave and reenter the survey without losing progress; once the survey is submitted, the code can no longer be used to access the survey thus preventing duplicate entries.

Care aides (unregulated staff) will be scheduled to meet in a private and quiet space with a trained data collector via Microsoft Teams. The data collector will administer the surveys using Computer Assisted Personal Interviewing (CAPI) techniques; survey questions will be read to the participant in a structured interview format using standardized language, and the data collector will enter responses directly into the Nooro research platform. In the event of a staff member becoming upset during data collection, interviews will be paused, and a support protocol will be initiated, including the provision of a list of local mental health resources. The variation in data collection procedures between regulated staff and care aides is based on previous feasibility testing conducted by the TREC team which determined structured interviews are more effective than self-administered web-based surveys for care aides and can be conducted in less time [27].

### Facility- and Unit-Level Data

One facility profile survey will be completed for each participating LTC home, and unit profile surveys will be completed for each unit in the LTC home. These surveys collect data such as the size, ownership model, unit information (ie, number and size of resident care units), types of units (eg, dementia and psychiatric), human resources (eg, physicians and nurse practitioners), care staff complement (eg, type and number of staff on each unit on a given shift), quality improvement activities, and access to programs and services. These surveys will be completed by research staff with LTC administrators or their designate (eg, a director of care or unit manager) through Microsoft Teams during or shortly after the staff data collection period.

### Staff Data

Surveys or interviews can be completed during work time and will take approximately 35 minutes. Multimedia Appendix 2 details the measures that will be collected. Survey item totals varied by staff survey type: care aide surveys have 154-169 items depending on responses to adaptive questions and are answered continuously in the interview format; nurse surveys have 207-219 items across 50 pages; allied health surveys have 138-149 items across 37 pages; and manager surveys have 209-229 items across 53 pages. Items or scales were not randomly ordered. Regulated staff completing the web-based survey could review and change their answers if desired before submission. There are no automatic completion checks alerting participants if their web-based survey contains missed items. Data collectors administering the care aide survey are to check for completion, however, it is not obligatory for care aides to answer missing items.

### Data Collector Training

Training will be in accordance with TREC’s training program for quality assurance purposes and to ensure consistency. Data collectors will receive in-depth standardized CAPI and survey interview training, which will be done in collaboration with TREC’s field coordinator and the ARC LTC staff who have experience with implementing the TREC Survey.

Data collectors will participate in 3 training sessions followed by a quality assurance test. The 3 sessions will be 2-3 hours long and delivered in-person or using Microsoft Teams meetings. These web-based sessions will be recorded for onboarding additional staff at later points in time. The sessions will include didactic teaching, group discussion, and survey practice with peers. Topics will include project background, survey overview, CAPI administration, accessing the survey platform, consent process, shift schedules and resources, and strategies for challenging interviews (eg, technology issues, managing participant distress, keeping participants on track). In addition to survey practice during the 3 sessions, data collectors will be instructed to practice on their own or with friends and family.

During the quality assurance testing, each data collector will complete a minimum of 2 surveys. Data collectors must meet all criteria (eg, obtaining proper consent, reading questions verbatim, appropriate use of scales, proper flow, correctly inputting responses, acting professional, answering or navigating participant questions, managing technology issues, managing participant distress, keeping participants on track). If necessary, additional training will be provided to data collectors on an individual basis to ensure they are able to conduct quality interviews meeting all quality assurance criteria.

Upon completion of training, each data collector will complete at least 1 practice interview with an ARC LTC staff member. Data collectors must receive approval from the ARC LTC staff before being cleared to conduct interviews. Ongoing quality assessment will continue throughout the data collection phase to ensure proficiency in administering surveys using CAPI, consistency between data collectors, and consistency across time ensuring high-quality data.

### Ethical Considerations

Ethical approval for this protocol was obtained from the research ethics boards of the academic institutions of each of the investigators. The study was first reviewed and approved by the Research Ethics Board at Mount Saint Vincent University, Nova Scotia (2023-039), and then subsequently reviewed and approved by ethics boards at Dalhousie University, Nova Scotia (2023-6887); University of New Brunswick (2023-133); Memorial University of Newfoundland (2023.212 or 2024.1112); and Health Prince Edward Island (2023-10-19 or 2024-02-12). Care staff participation will be voluntary, and the information collected will be completely confidential. Data collection with care staff will be held in a private location and no names will be stored with study data. Potential participants will be sent a copy of the consent form prior to the interview, and it will be reviewed just prior to initiating an interview. Informed consent including the purpose of the study, approximate length of completion, data storage and privacy, research team, and ability to opt-out at any time prior to survey completion will be obtained prior to starting the survey. After each care aide survey, the data collector will complete an interview checklist which will capture feedback on the interview and rate its quality. Biweekly data quality reports will be compiled and the number of interviews completed and in progress, time to complete interviews, and occurrence and nature of missing data or extreme scores will be reviewed by investigators and staff. Any unexpected issues will be reviewed with data collectors on a regular basis. Data collectors may be observed conducting interviews for ongoing quality assurance purposes. Data are anonymized upon survey entry, and as such, there is no way to identify or remove participants' responses once the survey has been submitted. Study reports at the unit level will only be available if more than 8 staff from the care unit participate; in cases where fewer than 8 staff from 1 care unit participate, reports will only be available at the facility level to protect participants from being identified. No data will be presented unless the cell size is 5 or more. The identities of the LTC homes that participate will also be held in confidence; sites will only be described by their province, size, region, and language. All the data and the results will be kept indefinitely on a secure server in the Health Research Data Repository that resides in the Faculty of Nursing, University of Alberta. The nominated principal investigator JMK is the data custodian for these data. Contingent on subsequent ethics reviews, its access will be highly restricted. The Health Research Data Repository will support storage, data analysis, and access control for all ARC LTC team members and trainees. Research team members may access the data remotely using unique usernames and strong passwords. Users are unable to download any data or access the internet while logged into the repository. Identifiable data and personal information will be stored separately from their deidentified counterparts to ensure participant confidentiality. Any print documents will be stored securely in locked file cabinets by the project lead in each of the provinces. All staff who participate in the study will receive a CAD $10 (US $7.55) coffee gift card as a token of appreciation. These cards will be supplied to the facility liaison who will hand out cards to staff who agree to participate on survey completion. In addition, LTC homes that participate will be provided with a small stipend at the completion of the study to compensate for any work associated with their participation; the stipend will be based on the size of the home (ie, small, medium, or large). All staff will be given an opportunity to obtain a copy of the study’s findings.

### Knowledge Translation

As part of the feedback process, tailored reports will be generated for each individual care home. These care home reports will be shared with the home administrator. Staff participants will be given the opportunity to receive a generalized report on the study’s findings. If they wish to receive results, they will be asked at the end of the survey to provide an email address. Email addresses for feedback purposes will not be linked to any data provided by participants.

### Data Analysis Plan

To assess the quality of work life among staff working in LTC homes in Atlantic Canada, data will be analyzed by home, by province, and in combination to identify and assess associations, interactions, and predictors of health and work-life outcomes. No correction methods will be calculated. Regression analysis and statistical modeling techniques will be used to explain how work environments within LTC homes are impacting the quality of work life and health of staff. For example, is the work environment (an independent variable) correlated with job satisfaction and burnout? In what way do demographic variables, such as age, or English as a second language mediate the effect of work environment on quality of work life and intention to leave? Multimedia Appendix 3 shows the proposed statistical approaches. Multilevel modeling will be conducted to explore the complex interplay among characteristics at the micro (eg, LTC staff), meso (eg, unit and facility), and macro (eg, province) levels and their effects on indicators of quality of work life and health. Early careers researchers, trainees, and knowledge users will be involved in all aspects of data analysis to help build capacity for research in the LTC sector in Atlantic Canada.

## Results

Data collection occurred between November 2023 and June 2024 and is complete. Between July 2024 and September 2024, data were cleaned and organized for analysis. Data analysis is underway. Initially, individual reports will present descriptive data for each participating LTC home comparing their staff responses to mental health and well-being by occupational group with those of staff from all other facilities participating in their province. A series of three reports are proposed: (1) demographics and perceptions of work life (eg, burnout, job satisfaction, and intention to leave); (2) personal health and well-being (eg, general anxiety, posttraumatic stress disorder, and insomnia); and (3) organizational context (including the 10 dimensions of the ACT [44]; eg, leadership, culture, feedback, and staffing). Concurrent with this descriptive analysis, analytical analysis is planned for publication in peer-reviewed journals.

## Discussion

### Overview

This study aligns with Canadian and global priorities related to creating a sustainable health workforce and improving service delivery for those residing in LTC [47]. Specifically, our study builds on the influential research of the TREC group in Western Canada who developed and refined a comprehensive methodology for understanding the context of LTC and more notably, identifying solutions for enhancing the work environment and improving the quality of resident care [15,20]. Such work has been proven effective in enriching the work life of staff and improving system efficiency [15,16]. To date, a key finding of TREC’s work is the importance of organizational context to support the implementation of interventions to improve staff outcomes. Given the difference in the organization and delivery of LTC services across Canadian provinces [4], this study will generate much-needed data for enhanced and targeted planning and decision-making in the Atlantic LTC sector. Findings from this study will support a greater understanding of our LTC work environment and workforce, and to use these regional data to determine where knowledge users can introduce interventions to improve staff quality of work life.

### Expected Findings

Concerns about the LTC workforce have existed for some time, but these have heightened since the COVID-19 pandemic [41]. The pandemic brought unprecedented challenges to LTC and staff were forced to navigate these stressors while maintaining the highest standards of care under difficult and often unsafe conditions. We expect to verify the extent to which these conditions continue to impact staff’s health and well-being, including stress, anxiety, and quality of work life, and assess whether care aides continue to report a poorer quality of work life than other LTC staff as reported in a Nova Scotian study in 2021 [48]. This Nova Scotian study’s finding is significant as it underscores the distinct struggles care aides face in LTC. These struggles may stem from providing direct care to increasingly complex residents or they may be associated with undervaluing of their work within an LTC work environment (eg, low pay, limited education), or both.

Should our research findings, across LTC settings and geographical locations, be consistent with those of the study by Keefe et al [48], it will provide further evidence to advocate for tailored supportive interventions specifically designed for care aides. For instance, our findings could inform policies related to the minimum educational requirements of care aides, which are currently lacking in some Atlantic Canadian provinces. Research findings could also support the need for job redesign, particularly efforts to provide care aides with more formal interaction within the workplace. Enhancing care aides’ inclusion into decision-making processes, providing mentorship programs, or enriching team-based care in a way that acknowledges and values the perspective that care aides bring will enable greater connection to their workplace [16,49,50]. Such approaches could recognize the vital contributions of care aides, affirming their roles as essential members of care teams, and enhancing their status within the workplace.

We are confident that our study will provide data to assess recent policy initiatives within Atlantic Canada regarding the recruitment of internationally educated nurses to address the staffing shortage in LTC. These efforts include targeted recruitment campaigns, streamlined credentialing processes, and both professional and personal support programs. In the short term, these efforts appear to be successful as evidenced by the increasing number of nurses who have migrated to the region from targeted countries. However, despite these short-term successes, little is known about the long-term sustainability and effectiveness of these recruitment strategies, especially in terms of retention and overall job satisfaction. This gap is important because the long-term success of these initiatives is crucial for ensuring that Atlantic Canada can maintain a stable and skilled health care workforce in the face of ongoing human resource challenges. Our data will offer valuable insights not only into the work patterns of these migrant workers but also into their work integration experiences, including job satisfaction, relationships with colleagues, and their intentions to leave their current roles. Such information will be crucial for understanding the true impact of international recruitment efforts and for identifying areas where improvements can be made to enhance retention and overall workforce stability in our region.

It is also expected that our findings will be able to provide recommendations for how to address the deficits in the LTC system that have been exposed during the COVID-19 pandemic [51]. As the Atlantic provinces are home to the oldest population in Canada, policy makers require region-specific data to ensure sustainable and quality LTC is available for those who need it. Efforts to provide quality LTC must recognize that work conditions are care conditions, and consider the context where care takes place. The results of this study will support workforce planning and provide much-needed data on the LTC sector’s current and future capacity. Study findings promise to identify elements in the work environment that are amendable to change and enable policy makers to develop strategic responses needed to support care staff, enhance worker health and well-being, and ultimately create the conditions that maximize staff’s ability to provide quality care.

### Limitations

The study has a number of limitations. While it is anticipated that there will be great interest among the LTC sector to participate in this research, it is possible that some homes may still be recovering from the pandemic or have human resources challenges that preclude them from participating in the study. We are using a stratified random sample to ensure provincial representation but the strata used in the sampling frame need to be adapted to reflect the uniqueness of each province. For example, language (French and English) is a stratum used only in the sample of LTC homes in New Brunswick, and the ownership model (ie, public, private for-profit, and not-for-profit) is only being used with the LTC homes in Prince Edward Island and Nova Scotia. While the decision to modify these strata was made to ensure adequate representation of subgroups within each province, we recognize the need for our data analysis procedures to account for these differences. We will perform rigorous comparisons between provincial data to identify and account for differences in strata. If necessary, we will use statistical techniques such as multilevel modeling to account for differences across provinces. We will also acknowledge any potential threats to external validity that may arise as a result of our sampling plan during our dissemination activities.

## Appendix

Multimedia Appendix 1 Checklist for Reporting Results of Internet E-Surveys (CHERRIES).

Multimedia Appendix 2 Overview of instruments used to measure micro- and meso-level concepts in the Atlantic Canada long-term care study: Translating Research in Elder Care survey.

Multimedia Appendix 3 Analytic Plan.

Multimedia Appendix 4 Peer review report.

## Abbreviations

ACT: Alberta Context Tool
ARC LTC: Atlantic Research Collaboration on Long Term Care
CAPI: Computer Assisted Personal Interviewing
CHERRIES: Checklist for Reporting Results of Internet E-Surveys
LTC: long-term care
TREC: Translating Research in Elder Care
HRDR: Health Research Data Repository

## Data Availability

The datasets generated or analyzed during this study are not publicly available due to limited access to the University of Alberta Health Research Data Repository where they are housed. Data are available from the corresponding author on reasonable request.
