# Supplementary material for: Examining Quality of Work Life in Atlantic Canadian Long-Term Care Homes: Protocol for a Cross-Sectional Survey Study
Source: JMIR Res Protoc. 2025 Mar 31;14:e66338. doi: 10.2196/66338 (PMC11997533; doi:10.2196/66338)
Supplement: Multimedia Appendix 1 [file resprot_v14i1e66338_app1.pdf]

Multimedia Appendix 1. Checklist for Reporting Results of Internet E-Surveys (CHERRIES)

Eysenbach G Improving the Quality of Web Surveys: The Checklist for Reporting Results of Internet E-Surveys (CHERRIES) J Med Internet Res 2004;6(3):e34 doi: 10.2196/jmir.6.3.e34. PMID: 15471760 PMCID: 1550605

Used with Permission: "Except where otherwise noted, articles published in the Journal of Medical Internet Research are distributed under the terms of the Creative Commons Attribution License (http://www.creativecommons.org/licenses/by/2.0/), which permits unrestricted use, distribution, and reproduction in any medium, provided the original work is properly cited.

Table S1. Checklist for Reporting Results of Internet E-Surveys (CHERRIES)

	**Checklist for Reporting Results of Internet E-Surveys (CHERRIES)**		
Item Category	***Checklist Item***	***Explanation***	***Application in Paper***
**Design**			
	Describe survey design	Describe target population, sample frame. Is the sample a convenience sample? (In "open" surveys this is most likely.)	Not a convenience sample: Targeted occupational groups in LTC who met specific eligibility criteria
**IRB (Institutional Review Board) approval and informed consent process**			
	IRB approval	Mention whether the study has been approved by an IRB.	Table 4
	Informed consent	Describe the informed consent process. Where were the participants told the length of time of the survey, which data were stored and where and for how long, who the investigator was, and the purpose of the study?	Lines 318 in data collection section and Lines 395-402 in the Ethical Considerations section
	Data protection	If any personal information was collected or stored, describe what mechanisms were used to protect unauthorized access.	Any identifying information on the participant was removed after the data cleaning

			process and not included in the final data records See lines 411-412
**Development and pre-testing**			
	Development and testing	State how the survey was developed, including whether the usability and technical functionality of the electronic questionnaire had been tested before fielding the questionnaire.	Survey is an established survey used in multiple waves of study. It was applied in Atlantic Canada for first time, French version was tested (See Translation section Lines 278-301)
**Recruitment process and description of the sample having access to the questionnaire**			
	Open survey versus closed survey	An "open survey" is a survey open for each visitor of a site, while a closed survey is only open to a sample which the investigator knows (password-protected survey).	Closed
	Contact mode	Indicate whether or not the initial contact with the potential participants was made on the Internet. (Investigators may also send out questionnaires by mail and allow for Web-based data entry.)	No contact on the internet – it is a closed survey. See detail in methods under Staff sample/sampling procedures Lines 230--263
	Advertising the survey	How/where was the survey announced or advertised? Some examples are offline media (newspapers), or online (mailing lists – If yes, which ones?) or banner ads (Where were these banner ads posted and what did they look like?). It is important to know the wording of the announcement as it will heavily influence who chooses to participate. Ideally the survey announcement should be published as an appendix.	Closed survey – only those employees eligible were invited to participate. Emails and posters were circulated in the randomly selected LTC homes
**Survey administration**			

	Web/E-mail	State the type of e-survey (eg, one posted on a Web site, or one sent out through e-mail). If it is an e-mail survey, were the responses entered manually into a database, or was there an automatic method for capturing responses?	See data collection procedures (lines 303-334)
	Context	Describe the Web site (for mailing list/newsgroup) in which the survey was posted. What is the Web site about, who is visiting it, what are visitors normally looking for? Discuss to what degree the content of the Web site could pre-select the sample or influence the results. For example, a survey about vaccination on a anti-immunization Web site will have different results from a Web survey conducted on a government Web site	N/A was a data platform
	Mandatory/voluntary	Was it a mandatory survey to be filled in by every visitor who wanted to enter the Web site, or was it a voluntary survey?	Voluntary
	Incentives	Were any incentives offered (eg, monetary, prizes, or non-monetary incentives such as an offer to provide the survey results)?	$10 coffee card for individuals (see lines 430-436)
	Time/Date	In what timeframe were the data collected?	Each LTC home had 1-2 weeks to collect data during the study data collection Nov-June 2023-2024
	Randomization of items or questionnaires	To prevent biases items can be randomized or alternated.	No
	Adaptive questioning	Use adaptive questioning (certain items, or only conditionally displayed based on responses to other items) to reduce number and complexity of the questions.	Yes

	Number of Items	What was the number of questionnaire items per page? The number of items is an important factor for the completion rate.	See lines 482-489
	Number of screens (pages)	Over how many pages was the questionnaire distributed? The number of items is an important factor for the completion rate.	See lines 346-354
	Completeness check	It is technically possible to do consistency or completeness checks before the questionnaire is submitted. Was this done, and if "yes", how (usually JAVAScript)? An alternative is to check for completeness after the questionnaire has been submitted (and highlight mandatory items). If this has been done, it should be reported. All items should provide a non-response option such as "not applicable" or "rather not say", and selection of one response option should be enforced.	Self-directed surveys (nurses, managers and Allied Health) do not have a completeness check. Surveys that were administered (Care Aides) the interviewer would check for its completeness. All participants can opt to not answer questions.
	Review step	State whether respondents were able to review and change their answers (eg, through a Back button or a Review step which displays a summary of the responses and asks the respondents if they are correct).	Yes
**Response rates**			
	Unique site visitor	If you provide view rates or participation rates, you need to define how you determined a unique visitor. There are different techniques available, based on IP addresses or cookies or both.	N/A closed survey
	View rate (Ratio of unique survey visitors/unique site visitors)	Requires counting unique visitors to the first page of the survey, divided by the number of unique site visitors (not page views!). It is not unusual to have	N/A closed survey

		view rates of less than 0.1 % if the survey is voluntary.	
	Participation rate (Ratio of unique visitors who agreed to participate/unique first survey page visitors)	Count the unique number of people who filled in the first survey page (or agreed to participate, for example by checking a checkbox), divided by visitors who visit the first page of the survey (or the informed consents page, if present). This can also be called "recruitment" rate.	60% of eligible was the goal<br><br>Total response rate was 61.6% across all provinces/staff groups.
	Completion rate (Ratio of users who finished the survey/users who agreed to participate)	The number of people submitting the last questionnaire page, divided by the number of people who agreed to participate (or submitted the first survey page). This is only relevant if there is a separate "informed consent" page or if the survey goes over several pages. This is a measure for attrition. Note that "completion" can involve leaving questionnaire items blank. This is not a measure for how completely questionnaires were filled in. (If you need a measure for this, use the word "completeness rate".)	Specific criteria are used in order to be eligible to complete both the online survey with care aides administered by data collectors and for other employee groups the self-administered online survey. The Data Management Committee reviewed all missing data and very few were incomplete.
**Preventing multiple entries from the same individual**			
	Cookies used	Indicate whether cookies were used to assign a unique user identifier to each client computer. If so, mention the page on which the cookie was set and read, and how long the cookie was valid. Were duplicate entries avoided by preventing users access to the survey twice; or were duplicate database entries having the same user ID eliminated before analysis? In the latter case, which entries were kept for analysis (eg, the first entry or the most recent)?	NO cookies were used closed platform only available to eligible participants who agree to complete the survey

	IP check	Indicate whether the IP address of the client computer was used to identify potential duplicate entries from the same user. If so, mention the period of time for which no two entries from the same IP address were allowed (eg, 24 hours). Were duplicate entries avoided by preventing users with the same IP address access to the survey twice; or were duplicate database entries having the same IP address within a given period of time eliminated before analysis? If the latter, which entries were kept for analysis (eg, the first entry or the most recent)?	N/A see above
	Log file analysis	Indicate whether other techniques to analyze the log file for identification of multiple entries were used. If so, please describe.	No
	Registration	In “closed” (non-open) surveys, users need to login first and it is easier to prevent duplicate entries from the same user. Describe how this was done. For example, was the survey never displayed a second time once the user had filled it in, or was the username stored together with the survey results and later eliminated? If the latter, which entries were kept for analysis (eg, the first entry or the most recent)?	Each participant was given a unique code. The Quality Assurance Committee reviews any surveys that had the same first and last name and the same DOB. If this occurs the record will be removed. This information was deleted from the data file onoce quality assurance was complete.
**Analysis**			
	Handling of incomplete questionnaires	Were only completed questionnaires analyzed? Were questionnaires which terminated early (where, for example, users did not go through all questionnaire pages) also analyzed?	Only questionnaires that had less than 15% missing and most scales completed will be retained
	Questionnaires submitted with	Some investigators may measure the time people needed	N/A

	an atypical timestamp	to fill in a questionnaire and exclude questionnaires that were submitted too soon. Specify the timeframe that was used as a cut-off point, and describe how this point was determined.	
	Statistical correction	Indicate whether any methods such as weighting of items or propensity scores have been used to adjust for the non-representative sample; if so, please describe the methods.	None – Our sample is based on a stratified random selection. (see lines 209-225)
