# Supplementary material for: Examining Quality of Work Life in Atlantic Canadian Long-Term Care Homes: Protocol for a Cross-Sectional Survey Study
Source: JMIR Res Protoc. 2025 Mar 31;14:e66338. doi: 10.2196/66338 (PMC11997533; doi:10.2196/66338)
Supplement: Multimedia Appendix 2 [file resprot_v14i1e66338_app2.pdf]

Multimedia Appendix 2

Figure S1 *Key content areas of the survey*

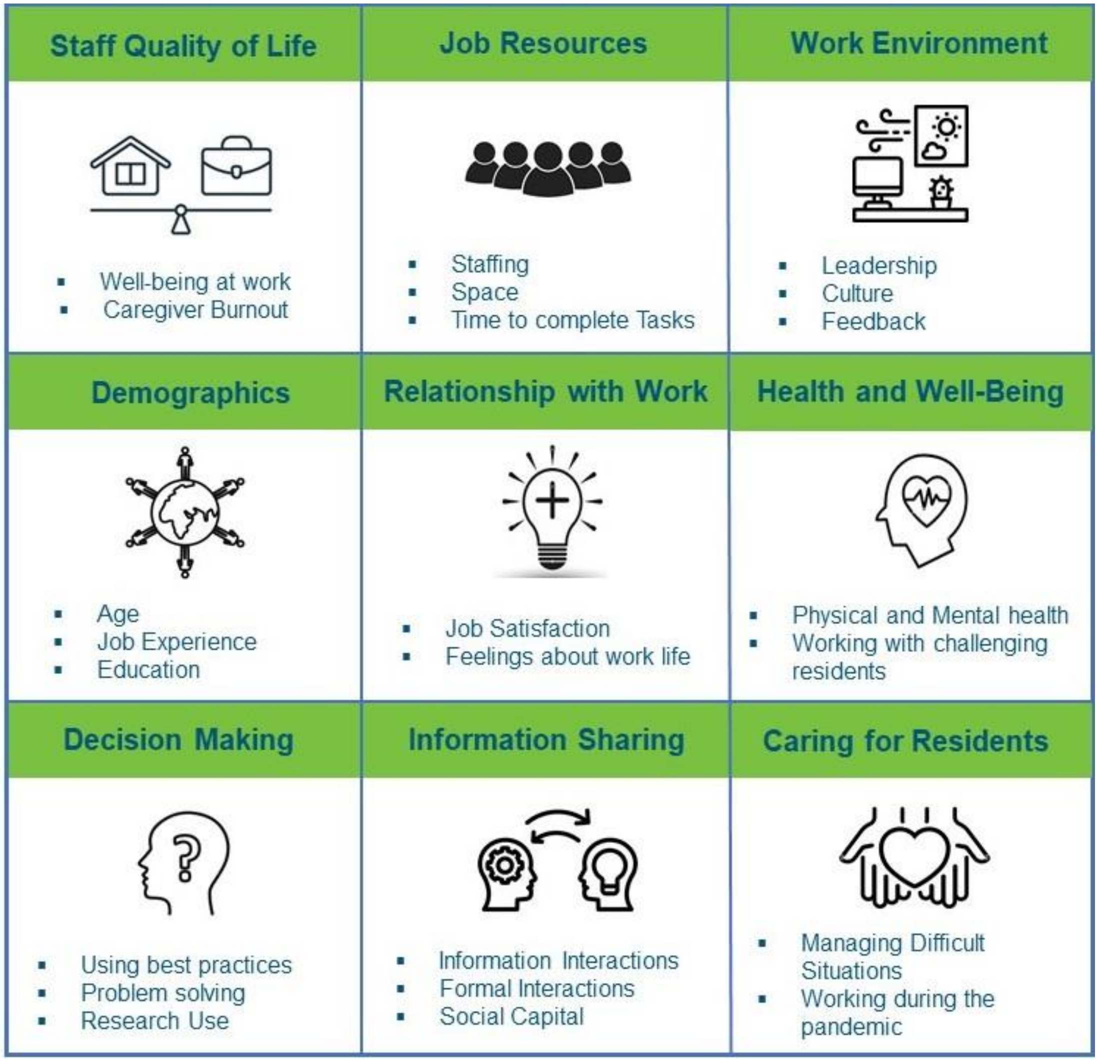

Table S1

Overview of Instruments Used to Measure Micro and Meso Concepts in the Atlantic Canada Long Term-Care – TREC Survey

**Instrument**	**Description**	**Scoring**	**Psychometric Properties**	**French Translation**
**MICRO**				
**Michigan Organizational Assessment Questionnaire (MOAQ); Job Satisfaction Subscale (Modified version)**	Global measure of affective components (i.e., feelings) about one's job.	3-items scored on a 5-point Likert scale (1 = strongly disagree to 5 = strongly agree).<br>Overall score derived by computing the mean of the three items (range 1 to 5) with higher values indicating higher job satisfaction.	Scale adapted for care aides in nursing homes and pilot tested in an Ontario sample in 2014 and field tested in Year 1 of TREC 2.0.<br>Cronbach alpha .82 in study of allied healthcare providers in 77 LTC facilities.[1]	Translated in French and validated
**Maslach Burnout Inventory, General Survey (short form)**	Composed of 3 subscales: Exhaustion, Cynicism and Efficacy.	Each subscale contains 3 items scored on a 7-point frequency scale (0 = never to 6 = daily)<br>Mean score computed for each subscale.<br>Higher scores negative (more burnout)	Cronbach's alpha just below or exceeded 0.70 in study of 757 RNs and LPNs from 91 LTC facilities.[2]	French Version Previously validated
**Short Form-8 (SF-8)**	Composite of mental and physical well-being. Items measure 8 domains: physical functioning, role physical, bodily pain, general health, vitality, social functioning, role emotional, and mental health.	8 items scored on 5- or 6-point scales depending on item.<br>Each item assigned appropriate score according to algorithm based on USA population data.[3] Summary scores generated for physical and mental health with range of 0 to 100 (higher = better).	Differences in physical and mental health observed in a comparison study involving healthcare workers in nursing homes (n = 2,028) and pediatric hospitals (1,036) as well as normative data for the Canadian population.[4]	French Version Previously validated
**General Anxiety Disorder-7 (GAD-7)**	Screening tool for detecting presence and severity of generalized anxiety disorder.	7 items scored on 4-point frequency scale (0=not at all to 3=nearly every day).<br>Total summative score ranges from 0 to 21. Higher scores more severe anxiety.	Useful tool with strong criterion validity for identifying probable cases of generalized anxiety disorder. GAD-7 has excellent internal consistency (Cronbach $\alpha = .92$), good test-retest reliability (intraclass correlation = 0.83), and good procedural validity (intraclass correlation = 0.83).	French Version Previously validated

Instrument	Description	Scoring	Psychometric Properties	French Translation
**MICRO**				
**The Abbreviated PCL-C: The Post-Traumatic Check-list -6-item Civil-ian Version (PCL-C)**	6-item version of the PTSD-Checklist, a self-report measure that as-sesses presence and se-verity of PTSD symp-toms.	6 items scored on 5-point fre-quency scale (1 = not at all to 5 = extremely).<br>Summative scores range from 6 to 30. Scores of 14 or higher suggestive of difficulties with post-traumatic stress with fur-ther assessment warranted.	An abbreviated version of the 17-item Post-Traumatic Checklist – Civilian version was developed and vali-dated by Lang and Stein (2005) as a brief screening tool for PTSD in primary care.[5]	Translated in French and validated
**Short Professional Quality of Life Scale (ProQOL)**	9-item version of the ProQOL questionnaire designed to measure burnout, compassion fa-tigue. and compassion satisfaction in helping professionals (e.g., health care workers, teachers).	9 items scored on a 5-point fre-quency scale (1 = never to 5 = very often). Three items for each subscale (burnout, com-passion fatigue, compassion satisfaction). Scores for each subscale summed and then con-verted to t-scores.	Short version of ProQOL developed and initial psycho-metric testing by Galiana et al. (2020) with a sample of palliative care professionals (e.g., physicians, nurses, psychologists, nursing assistants, social workers) from Spain, Argentina, and Brazil.[6]	French Version Pre-viously validated
**Perceived Stress Scale (PSS-4)**	4-item tool measuring the extent an individual has perceived life as unpre-dictable, uncontrollable and overloading during last month.	4 items scored on a 5-point fre-quency scale (0 = never to 4 = very often).<br>Summative scores range from 0 to 16 with higher scores indi-cating situations in one's life appraised as more stressful.	Developed and initial psychometric testing by Cohen et al. (1983).[7]	Translated in French and validated
**Brief Resilience Scale (BRS)**	6-item tool measuring perceived ability to bounce back or recover from stress. Items 1, 3, and 5 are positively worded, and items 2, 4, and 6 are negatively worded. Possible score range from 1 (low resili-ence) to 5 (high resili-ence).	6 items scored on a 5-point agreement scale (1 = strongly disagree to 5 = strongly agree).<br>Mean score computed for items.<br>Higher scores indicate perceived ability to bounce back from or adapt to stress.	Developed and initial psychometric testing by Smith et al. (2008) with samples of students and patients (car-diac and chronic pain).[8]	French Version Pre-viously validated
**Task Left Undone**	Measures care related ac-tivities not performed by provider during last shift due to lack of time (e.g.,	10 items scored dichotomously (yes/no).<br>Score is number of 'yes' re-sponses.	List of items developed for TREC studies based on care aides' responses to an open-ended question.[9]<br>In cross-sectional TREC study involving 4,016 care	Translated in French and validated

Instrument	Description	Scoring	Psychometric Properties	French Translation
**MICRO**				
	nail care, mouth care, toileting).	Higher scores more activities not performed.	aides, those working in more favorable work environments were less likely to reported tasks left undone (OR 1.59, 95% CI 1.34-1.90).[10]	
**Tasks Rushed**	Measures care related activities that providers reported feeling rushed to complete during last shift (e.g., talking with residents, feeding, toileting)	7 items scored dichotomously (yes/no).<br>Score is number of 'yes' responses.<br>Higher scores, higher feeling of being rushed.	List of items developed for TREC studies based on care aides' responses to an open-ended question.[9]<br>In cross-sectional TREC study involving 4,016 care aides, those working in more favorable work environments were less likely to reported rushing to complete tasks (OR 1.66, 95% CI 1.38-1.99).[10]	Translated in French and validated
**Insomnia Severity Index**	Measures subjective symptoms and consequences of insomnia as well as degree of concern or distress evoked.	7 items scored on a 5-point rating scale.<br>Total summative score ranging from 0 to 28. Higher scores indicating more severe sleep problem.	Developed and initial psychometric testing by Bastien et al. (2001) with samples of insomnia patients.[11]<br>Instrument added to TREC suite of survey instruments for this study.	French Version Previously validated
**MESO**				
**Alberta Context Tool (ACT)**	Developed to measure the organizational context within healthcare settings in terms of 10 concepts: leadership, culture, evaluation, formal interactions, informal interactions, social capital, structural resources including 3 scales for organizational slack (staffing, space, time).	Number of items vary from 56 to 58 depending on healthcare setting.<br>Items scored on a 5-point Likert scale (1 = strongly disagree to 5 = strongly agree).<br>Scores for each concept derived by computing mean of the items (range 1 to 5) with higher scores indicating more favorable work environment .	Tool originally tested in acute care and then adapted for LTC and tested for reliability and validity with care aides.[12]<br>Cronbach's alpha exceeded 0.70 for 8 of 10 concepts in the care aide version and 9 of 10 concepts in the nurse version.<br>Confirmatory factor analyses of both versions demonstrated consistency of overall pattern of data tested with the structure hypothesized in developing the ACT.[13]	Translated in French and validated
**Unit Level Survey**	A 12-part survey including measures of unit type, unit clerk, physician and nurse practitioner interaction, staffing level, students, response to major near misses, organiza-	Categories vary across each part of the survey.	N/A	Not translated, verbally conducted in French if requested.

Instrument	Description	Scoring	Psychometric Properties	French Translation
**MICRO**				
	tional behaviour, physician accessibility and continuity, Unit-level learning from patient safety events, and Observable Indicators of Nursing Home Care Quality.			
**Facility Characteristics Survey**	A 10-part survey including facility information, unit information, allied services/programs offered, performance reports, medical care, access to specialized medical services, quality improvement activities, organization site, ownership model, staffing level.	Categories vary across each part of the survey.	N/A	Not translated, verbally conducted in French if requested.

References

1. Aloisio, L.D., Gifford, W.A., McGilton, K.S., Lalonde, M., Estabrooks, C.A., & Squires, J.E. (2018). Individual and organizational predictors of allied healthcare providers' job satisfaction in residential long-term care *BMC Health Services Research, 18*. Article 491. https://doi.org/10.1186/s12913-018-3307-3
2. Squires, J.E., Baumbusch, J., Demery Varin, M., MacDonald, I., Chamberlain, S., Bostrom, A-M., Thompson, G., Cummings, G., & Estabrooks, C.A. (2019). A profile of regulated nurses employed in Canadian long-term care facilities. *Canadian Journal of Aging, 38*(2), 130-142. https://doi.org/10.1017/S0714980818000478
3. Ware, J.E., Kosinski, M., Dewey, J.E., & Gandek, B. (2001). How to score and interpret single-item health status measures: A manual for users of the SF-8 health survey. QualityMetric Incorporated.
4. Hoben, M., Knopp-Sihota, J.A., Nesari, M., Chamberlain, S.A., Squires, J.E., Norton, P.G., Cummings, G.G., Stevens, B.J., & Estabrooks, C.A. (2017). Health of health care workers in Canadian nursing homes and pediatric hospitals: A cross-sectional study. *Canadian Medical Association Open, 5*(4), E791-E799. https://doi.org/10.9778/cmajo.20170080
5. Lang, A.J., & Stein, M.B. (2005). An abbreviated PTSD checklist for use as a screening instrument in primary care. *Behaviour Research and Therapy, 43*, 585-594. https://doi.org/10.1016/j.brat.2004.04.005
6. Galiana, L., Oliver, A., Arena, F., De Dimone, G., Tomás, J.M., Vidal-Blanco, G., Muňoz-Martinez, I., & Sansŏ, N. (2020). Development and validation

of the *Short Professional Quality of Life Scale* based on versions IV and V of the *Professional Quality of Life Scale*. *BMC Health and Quality Life Outcomes, 18*. Article 364. https://doi.org/10.1186/s12955-020-01618-3
7. Cohen, S., Kamarch, T., & Mermelstein, R. (1983). A global measure of perceived stress. *Journal of Health and Social Behavior*, *24*(4), 385-396.
8. Smith, B.W., Dalen, J., Wiggins, K., Tooley, E., Christopher, P., & Bernard, J. (2008). The Brief Resilience Scale: Assessing the ability to bounce back. *International Journal of Behavioral Medicine*, *15*, 194-200. https://doi.org/10.1080/10705500802222972
9. Knopp-Sihota, J., Niehaus, L., Squires, J.E., Norton, P.G., & Estabrooks, C.A. (2015). Factors associated with rushed and missed resident care in western Canadian nursing homes: A cross-sectional survey of health care aides. *Journal of Clinical Nursing*, *24*(19-20), 2815-2825. https://doi.org/10.1111/jocn.12887
10. Song, Y., Hoben, M., Norton, P., & Estabrooks, C.A. (2020). Association of work environment with missed and rushed care tasks among care aides in nursing homes. *JAMA Network Open*, *3*(1), Article e1920092. https://doi.org/10.1001/jamanetworkopen.2019.20092
11. Bastien, C.H., Valliėres, A., & Morin, C.M. (2001). Validation of the Insomnia Severity Index as an outcome measure for insomnia research. *Sleep Medicine*, *2*(4), 297-307. https://doi.org/10.1016/S1389-9457(00)00065-4
12. Estabrooks, C.A., Squires, J.E., Cummings, G.G., Birdsell, J.M., & Norton, P.G. (2009). Development and assessment of the Alberta Context Tool. *BMC Health Services Research, 9*. Article 234. https://doi.org/10.1186/1472-6963-9-234
13. Squires, J.E., Hutchinson, A., Hayduk, L., Cranley, L.A., Cummings, G., Norton, P.G., & Estabrooks, C.A. (2014). *Alberta Context Tool User Manual*. Knowledge Utilization Studies Program, Faculty of Nursing, University of Alberta, Edmonton, Canada (ISBN: 978-1-55195-340-3).
