# Supplementary material for: Examining Quality of Work Life in Atlantic Canadian Long-Term Care Homes: Protocol for a Cross-Sectional Survey Study
Source: JMIR Res Protoc. 2025 Mar 31;14:e66338. doi: 10.2196/66338 (PMC11997533; doi:10.2196/66338)
Supplement: Multimedia Appendix 3 [file resprot_v14i1e66338_app3.pdf]

Multimedia Appendix 3

Data Analytic Plan for Atlantic Canada Long-term Care Study

Steps	Statistical Approaches
**Data Quality**: Item-level examination of data for the presence and nature of missing data, extreme scores (i.e., outliers), or other indicators of problematic variables (e.g., lack of variability).	Descriptive Statistics (numerical and graphic)
**Psychometric Analysis, Reliability**: Examination of internal consistency of scores for multi-item measurement instruments and comparison of results to those reported in previous studies. If acceptable, computation of subscale and total scores.	Cronbach's Alpha<br>Inter-Item Correlations
**Summarize Characteristics of Study Sample:** Creation of Summary Tables and Figures depicting the characteristics of participating facilities, units, and regulated & unregulated staff.	Descriptive Statistics (numerical and graphic)
**Summarize Key Study Variables**: Computation of statistics of central tendency, variability, and shape for measures of:<br>- Health = Mental & Physical Health, Anxiety, Post Traumatic Stress Disorder, Stress, Insomnia<br>- Wellness = Quality of Life, Resilience<br>- Quality of Work life = Job Satisfaction, Burnout, Tasks Left Undone, Tasks Rushed<br>- Organizational Context = Leadership, Culture, Interactions, Resources, Organizational Slack	Descriptive Statistics (numerical and graphic)
**Psychometric Analysis, Validity:** Examination of strength and direction of correlation coefficients among key variables for evidence of convergent and discriminant validity. Validation of the factor structure of measurement instruments through confirmatory factor analysis.	Bivariate Correlations<br>Confirmatory Factor Analysis
**Comparative Analysis:** Examination of differences in the indicators of the health, wellness, and quality of work life by:<br>- Size of Facility: Small, Medium, Large<br>- Ownership: Private versus Public<br>- Type of Provider: Regulated versus Unregulated	ANOVA<br>MANOVA
**Regression Analysis:** Investigation of meso- and micro-level variables that help explain variance in the health, wellness, and quality of work life of LTC staff	Multiple Regression<br>Statistical Modeling
**Exploratory Analysis:** Examination of the feasibility and process for linking ARC-TREC and interRAI data to investigate the effect of the LTC staff's work environment and quality of work life on resident outcomes.	Exploratory Analysis
